# Influence of the deviated center of rotation on the range of motion after cervical disc arthroplasty –an in vivo study with a minimum of 10-year follow-up

**DOI:** 10.1186/s12891-022-06041-x

**Published:** 2023-02-01

**Authors:** Kai Yan, Zhan Shi, Da He, Bo Liu, Bin Xiao, Qilong Wang, Wei Tian

**Affiliations:** Department of Spine Surgery, Beijing Jishuitan Hospital, No. 31, Xinjiekou East St, Xicheng District, Beijing, 100035 China

**Keywords:** Bryan cervical artificial discs, Range of motion, Center of rotation, Long-term follow-up

## Abstract

**Background:**

Short-term researches have studied the change of the center of rotation (COR) after the Bryan Cervical disc arthroplasty (CDA). But there is a lack of long-term studies focusing on the location of COR and its influence after surgery.

**Methods:**

Clinical and radiographic materials of patients who received CDA were retrospectively reviewed. Written informed consents were obtained. Clinical outcome was accessed by Japanese Orthopaedic Association (JOA), Neck Disability Index (NDI), and Odom’s scale. Radiographic evaluation underwent before surgery, at early (3 months) follow-up and final (minimal 10 years) follow-up. The ROM of the global cervical spine and index level, the functional spine unit (FSU) angle and C2-C7 angle were measured. COR was identified and its coordinates were calculated. The absolute change of COR-x and COR-y were compared in subgroup analysis.

**Results:**

Sixty patients were included, with an average age of 55.9 ± 8.1 years old. The mean duration of follow up was 135.1 ± 16.1 (120–155) months. JOA, NDI and Odom’s scale showed significant improvements at 10 years after surgery. The COR of index level was located in the posterior superior half of the caudal vertebral body. Following the implant of Bryan Disc, the COR shifted forward and downward. During the 10-year follow-up, the location of COR remained stable. ROM at the index level decreased from 10.6 ± 4.0° preoperatively to 9.3 ± 4.0° at the early follow-up (*p* = 0.03). The ROM at the index level remained unchanged from early follow-up to the final follow-up (9.3 ± 4.0° vs 9.5 ± 5.2°, *p* = 0.80). In subgroup analysis, larger changes of both COR-x and COR-y were related with decreased ROM.

**Conclusions:**

Our study illustrated that Bryan CDA could achieved favorable clinical and radiographic outcome over a minimal 10-year follow-up. The reduction of the flexion-extension ROM may be correlated with a more deviated postoperative COR. More attention should be paid to preoperative design and intraoperative technique to obtain a more native COR.

## Introduction

Symptomatic cervical degenerative disc disease (CDDD) is a common condition affecting the population in various age groups [1–4]. Although anterior cervical discectomy and fusion (ACDF) for CDDD has achieved satisfactory clinical outcomes [5], it inevitably leads to decreased cervical range of motion (ROM), and is hypothesized to accelerate the progressive degeneration of adjacent segments [4, 6]. Cervical disc arthroplasty (CDA), as the most representative cervical non-fusion technique, is an alternative to ACDF in the treatment of cervical degenerative disc disease. It could maintain the normal disc height, and preserve the segmental motion after surgery [7].

The principal goal of CDA is to restore the physiologic kinematics at the index level and the ideal CDA prosthesis should achieve this goal by restoring physiologic quantity and quality of motion. And Bryan disc was the most widely used and researched CDA prosthesis in the world. In previous literature about CDA, many studies have proven that the clinical outcomes were satisfactory, and the ROM, the quantity of motion, was maintained [8–10]. And there are some studies focusing on the location of the flexion-extension center of rotation (COR) as a measure of motion quality, including researches about the change of the COR after the Bryan CDA [6, 11] with a relatively short follow-up. But there is a lack of long-term studies focusing on the location of COR and its influence after the Bryan CDA. Thus, the purpose of present in vivo study was to evaluate the long-term clinical outcome, illustrate the deviation of COR after the Bryan CDA and its possible influence on cervical range of motion. Our hypotheses were that deviation of COR may have a negative influence on the cervical ROM.

## Materials and methods

### Patient population

We retrospectively enrolled 83 consecutive cases who underwent single-level cervical arthroplasty for cervical disc degenerative disease between January 2008 and January 2012. Written informed consents were obtained before surgery for the possibility of using their charts and radiographs. All surgeries were accomplished by a single surgeon (WT).

### Inclusion and exclusion criteria

The inclusion criteria were as follows: 1) diagnosis of single-level cervical disc degenerative disease, presented with either radiculopathy or myelopathy, or both; 2) age > 18 years; 3) no response to no-operative management for more than 6 weeks; 4) complete follow-up data; and 5) a preoperative ROM at the index level > 3°. The exclusion criteria involved: 1) patients with traumatic or infectious pathology, 2) previous cervical spine surgery, 3) diagnosis of multilevel cervical disc degenerative disease, 4) radiographically instability or loss of ROM (< 3°), severe facet degeneration, potential infection, poor bone quality or neoplasia, and 5) high level (grade III or IV [12]) heterotopic ossification at the final follow-up.

### Surgical procedure

After general anesthesia, the patient was placed in a supine position through a standard anterior cervical approach according to Smith–Robinson. After confirming the index cervical level by intraoperative fluoroscopy, a complete discectomy was performed. An accurate decompression of the spinal cord and nerve roots was obtained. After the vertebral endplate was polished, the proper size Bryan artificial disc (Medtronic Sofamor Danek USA, Inc) was implanted.

### Clinical evaluation

Before surgery and at last follow-up, clinical outcome was accessed by Japanese Orthopaedic Association (JOA) score for functional recovery, Neck Disability Index (NDI) for neck pain/disability, and Odom’s scale for overall efficacy. Each evaluation was performed by two separate doctors, and if disagreement was met, an advice from a third senior doctor would be sought.

### Radiographic evaluation

**Fig. 1:**
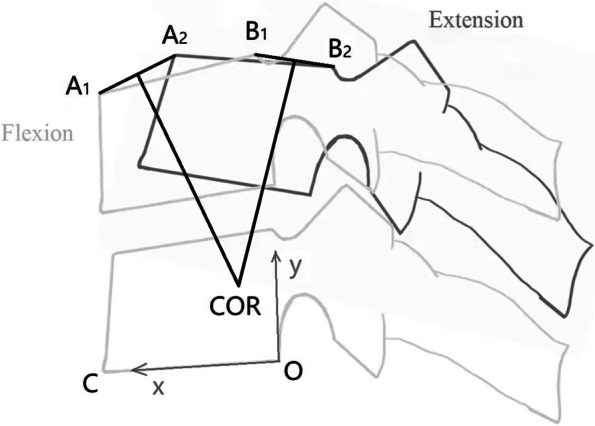
Schematic diagram showing the determination of the COR and calculation of its coordinates

Radiographic evaluation included lateral neutral and flexion-extension cervical radiographs before surgery, at early (3-month) follow-up and final follow-up. The ROM of the overall cervical spine (C2-7) and index level were measured on lateral flexion-extension radiographs according to the Cobb method [13]. The ROM of less than 3 degree was considered loss of mobility. COR was identified and its coordinates were calculated by the method shown in Fig. 1, and this methodology was validated in previous literature [14–17]: the line A1A2 connects anterior superior corner of the cranial vertebra in flexion (point A1) and that point in extension (point A2). The line B1B2 connects posterior superior corner of the cranial vertebra in flexion (point B1) and that point in extension (point B2). The intersection of perpendicular bisectors of line A1A2 and B1B2 is COR.

An orthogonal plane coordinate system is set up, the posterior inferior corner of the caudal vertebra (point O) is set as origin of coordinates, and the inferior endplate of the caudal vertebra (line OC) is set as x-axis. The y-axis was directed upward perpendicular to the x-axis. COR-x is positive in the anterior direction and COR-y is positive in the cranial direction. The coordinates of points A1, A2, B1 and B2 were measured in millimeter and expressed as (XA1, YA1), (XA2, YA2), (XB1, YB1) and (XB2, YB2). Analytical geometry method is used to calculate the coordinates of COR (COR-x, COR-y), as shown below:

$$\textrm{COR}_{\textrm{x}}=\left[0.5\left(\textrm{YB}1+\textrm{YB}2\right)+0.5\left(\textrm{XB}2-\textrm{XB}1\right)\left(\textrm{XB}1+\textrm{XB}2\right)/\left(\textrm{YB}2-\textrm{YB}1\right)-0.5\left(\textrm{YA}1+\textrm{YA}2\right)-0.5\left(\textrm{XA}2-\textrm{XA}1\right)\left(\textrm{XA}1+\textrm{XA}2\right)/\left(\textrm{YA}2-\textrm{YA}1\right)\right]/\left[\left(\textrm{XB}2-\textrm{XB}1\right)/\left(\textrm{YB}2-\textrm{YB}1\right)-\left(\textrm{XA}2-\textrm{XA}1\right)/\left(\textrm{YA}2-\textrm{YA}1\right)\right].$$

$$\textrm{COR}_{\textrm{y}}=0.5\left(\textrm{YA}1+\textrm{YA}2\right)+0.5\left(\textrm{XA}2-\textrm{XA}1\right)\left(\textrm{XA}1+\textrm{XA}2\right)/\left(\textrm{YA}2-\textrm{YA}1\right)-\textrm{COR}_{\textrm{x}}\left(\textrm{XA}2-\textrm{XA}1\right)/\left(\textrm{YA}2-\textrm{YA}1\right).$$

Then the COR-x value was normalized as percentage (%) of the inferior endplate length of the caudal vertebra body, and the COR-y value was normalized as percentage (%) of the posterior height of the caudal vertebra body.

Sagittal alignment included C2-C7 angle (the angle between lower endplate of C2 and the lower endplate of C7) and functional spine unit angle (FSU angle, the angle formed between the superior endplate of the cranial vertebra and the inferior endplate of the caudal vertebral.

### Subgroup analysis

Patients were separated into two groups according to the change of ROM at the index level. Cases with decreased ROM at last follow-up compared with the preoperative status were defined as group D-ROM. Cases with increased ROM at the index level were defined as group I-ROM. The absolute value of the change of COR-x and COR-y were compared between these two groups.

### Statistical analysis

Statistical analysis was performed using IBM SPSS Statistics 20.0 (SPSS, Chicago, IL, USA). All continuous variable data were assessed for normality using Shapiro–Wilk test statistics. The continuous variable data is given as the mean ± standard deviation. The Mann–Whitney U-test was used for non-normally distributed data. The student t-test was used to compare normally distributed data, and paired t-test for paired data. One-way repeated measures analysis of variance was used for comparisons involving 3 time points on the same subject to correct P value from paired t-test. The χ^2^ test or Fisher exact test was used to compare categorical data. Both of these two analyses were utilized to compare the difference of each parameter at different time-points, or between two subgroups. A p value < 0.05 was considered significant. All measurements were performed by two independent spine surgeons based on standardized criteria. The reliability of determining the location of COR was analyzed by inter- and intra-class correlation coefficient (ICC). The ICC values were graded using the following criteria: excellent for values in the 0.9–1.0 range, good for 0.7–0.89, fair/moderate for 0.50–0.69, low for 0.25– 0.49, and poor for 0.0–0.24.

## Results

### General information

We reviewed 83 patient’s charts, and finally 60 patients were included. Twelve patients were lost to follow up, 7 patients were excluded because of incomplete materials and 4 were due to the occurrence of grade III or grade IV heterotopic ossification (HO). The average age was 55.9 ± 8.1 (38-66) years old at last follow-up. It was composed of 24 females and 36 males, including 1 patient at the C3-4 index level, 13 patients at the C4-5 index level, 39 patients at the C5-6 index level and 7 patients at the C6-7 index level. The mean duration of follow up was 135.1 ± 16.1(120-155) months (Table 1).

**Table 1:** Demographic characteristics

Characteristics	Value
Patients (n)	60
Mean age (years), mean ± SD	55.9 ± 8.1
Sex (female/male)	24/36
Index level (n)	
C3–4	1
C4–5	13
C5–6	39
C6–7	7
Follow up (months), mean ± SD	135.1 ± 16.1

### Clinical outcomes

There was immediate relief of radiculopathy and/or myelopathy in all cases, with no operative or device-related complications over the follow-up. The JOA score of the overall group increased from 13.5 ± 3.3 before surgery to 16.5 ± 2.0 at the final follow-up (*p* < 0.001). The NDI declined from a preoperative score of 27.0 ± 10.0 to a 10-year postoperative score of 12.0 ± 8.0 (*P* < 0.001). Using Odom’s Scale, 91.7% of patients reported good or excellent outcome. And there was no infection, revision surgery or neurologically/technically related complications occurred upon minimal 10-year follow-up.

### Sagittal alignment

The C2-C7 angle before surgery was similar to that at early follow-up (15.7±10.5°vs 13.9 ±11.7°, *p*=0.229), and it remained stable during the 10-year follow-up (13.9 ±11.7° vs 14.8±10.7°, *p*=0.416). There was no significant change in mean FSU angle from the preoperative values to the postoperative values (1.2 ± 6.1° vs 1.8 ± 7.1°, *p* = 0. 268), and at the final follow-up, it remained stable (1.8±7.1° vs 0.2±6.9°, *p*=0.073). The FSU height value was normalized as percentage (%) of the posterior height of the caudal vertebra body. The FSU height before surgery was comparable to that at early follow-up (223.1 ± 12.9% vs 222.2 ± 18.4%, *p*=0.606), and it was maintained to the 10-year follow-up (222.2 ± 18.4% vs 220.5 ± 18.2%, *p*=0.197).

### Center of Rotation and its deviation

**Fig. 2:**
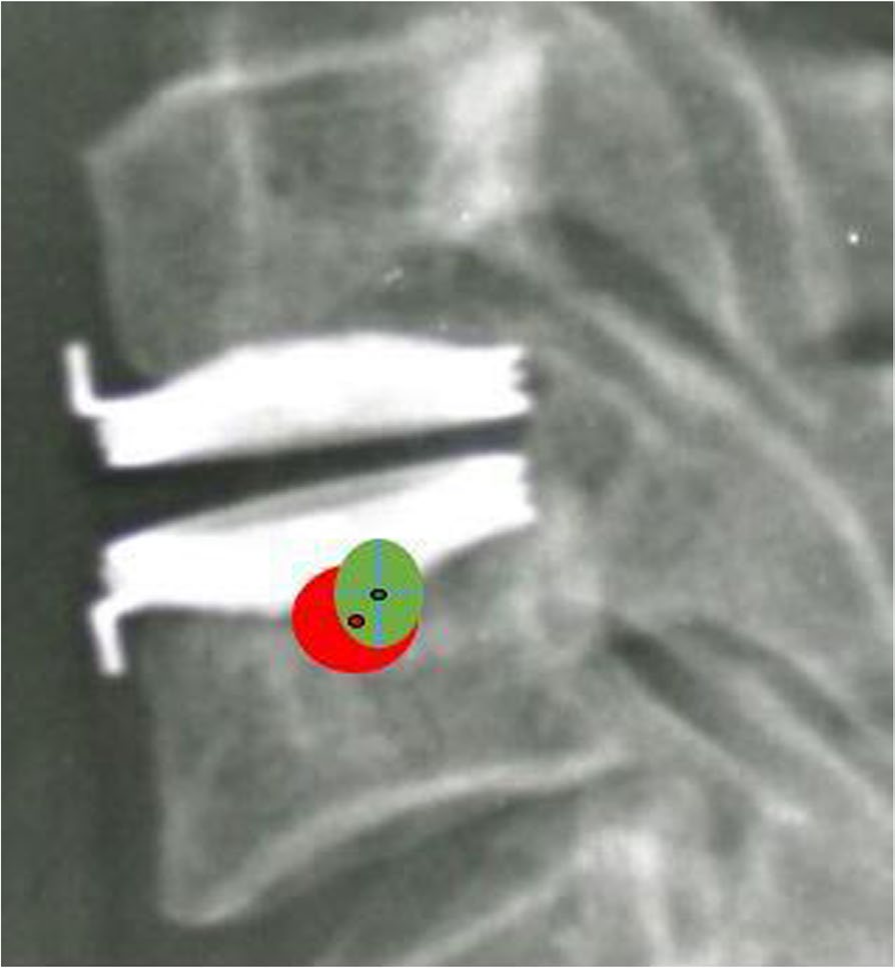
Graphic representation of COR before surgery (green field) and at the last follow-up (red field). Green dot represented mean COR-x/COR-y with green filed indicating 1 standard deviation before surgery. Red dot and field represented the mean and 1 standard deviation at the last follow-up. The COR position shifted more anterior and inferior after insertion of the Bryan disc

**Fig. 3:**
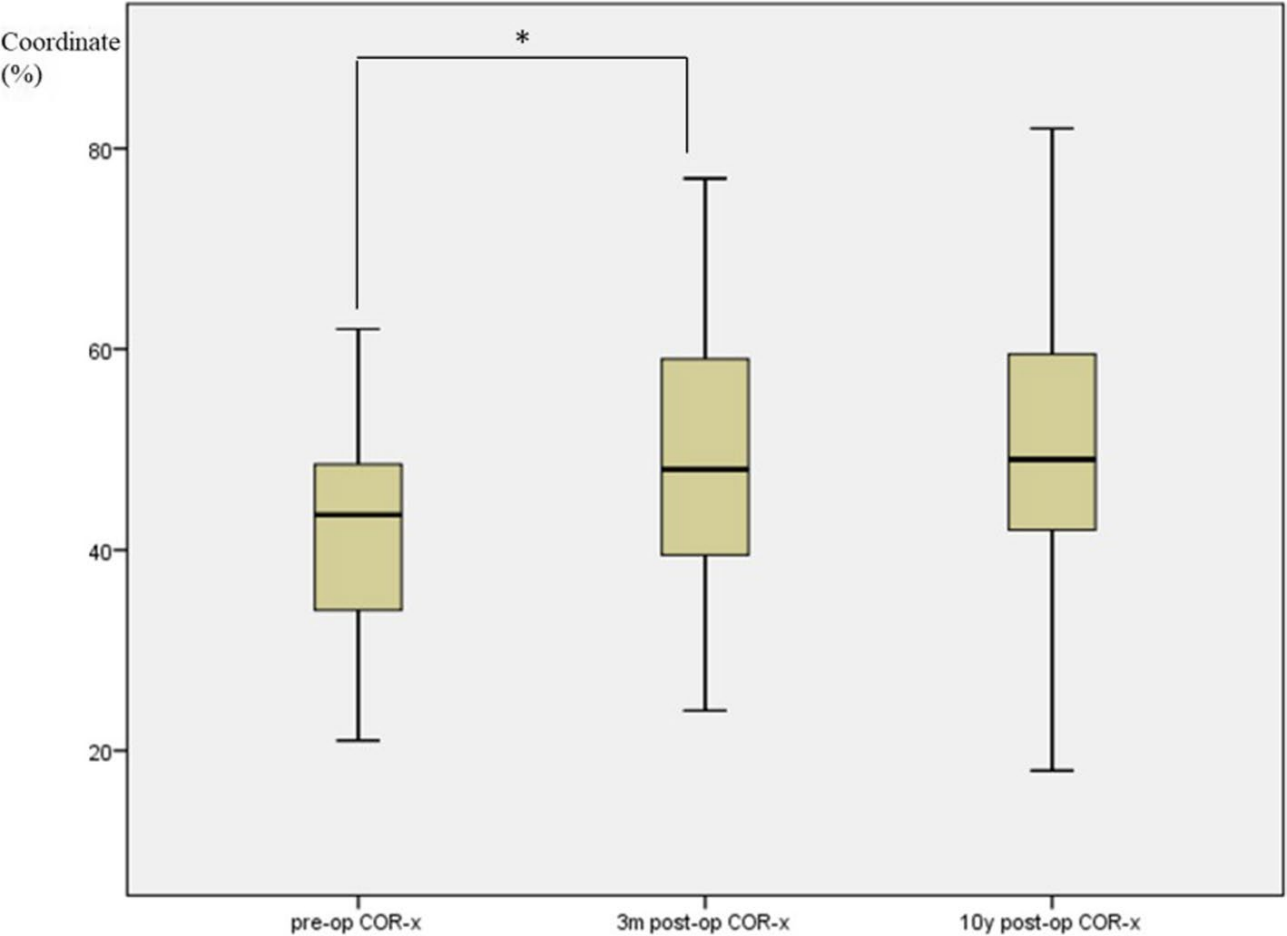
Boxplot shows the comparison of the COR-x before surgery, at 3 months follow-up, and at 10 years follow-up (* means significant difference)

**Fig. 4:**
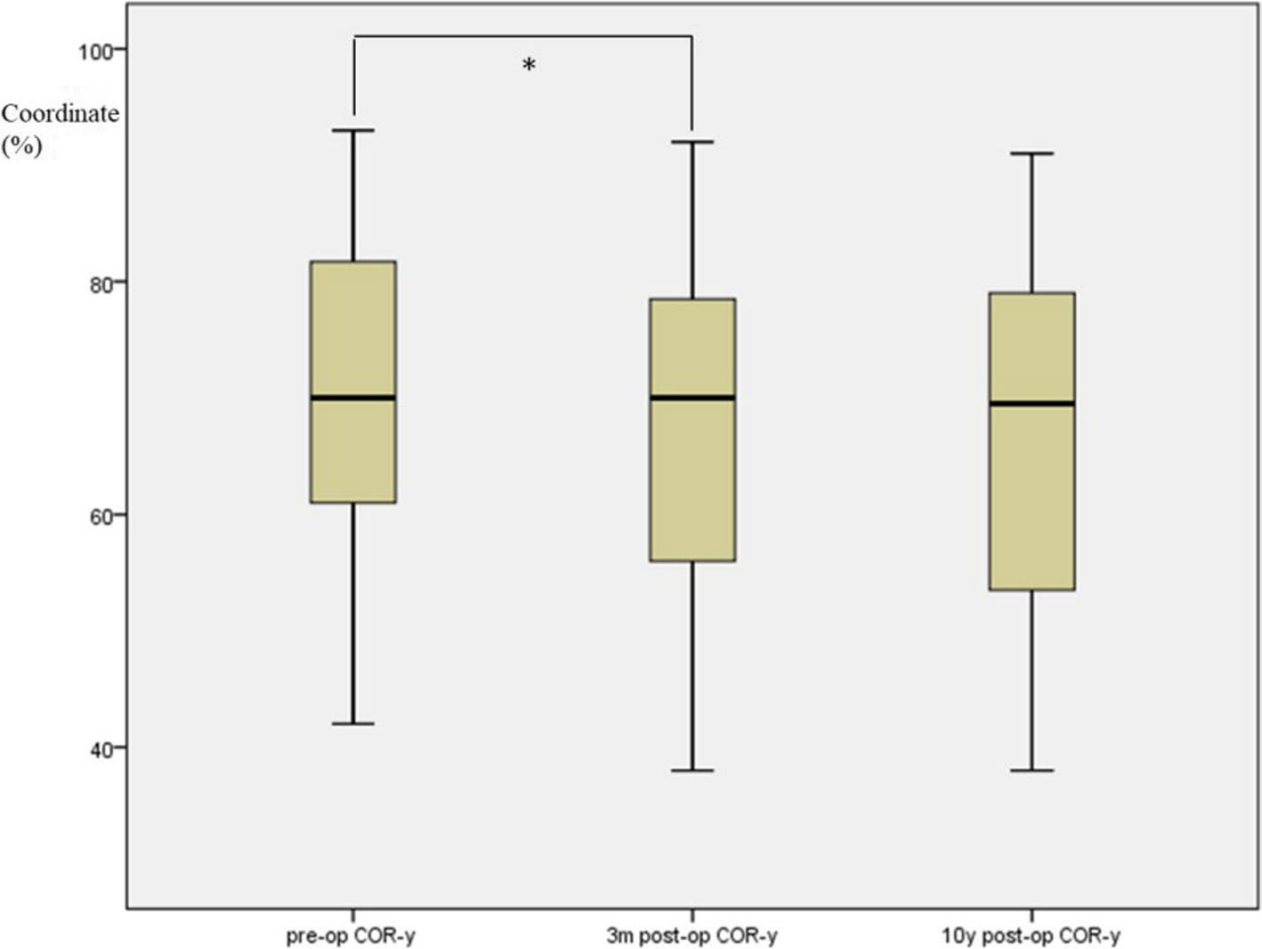
Boxplot shows the comparison of the COR-y before surgery, at 3 months follow-up, and at 10 years follow-up (* means significant difference)

Using ICC reliability statistics, the inter- and intra-observer reliability between two different spine surgeons were both higher than 0.89. The preoperative COR of the flexion-extension motion at index disc level was located in the posterior superior half of the caudal vertebral body. Following the implant of Bryan Disc, the COR shifted forward and downward (Fig. 2), as shown in the changes of COR coordinates. The COR-x increased from preoperative 42.4 ± 9.7% to 49.5 ± 14.1% at the early follow-up (*p* < 0.001), while COR-y decreased significantly after surgery from 70.6 ± 12.7% to 67.2 ± 14.0% (*p* = 0.003). At the 10-year follow-up, COR-x and COR-y showed no significant change compared with the early postoperative values (COR-x: from 49.5 ± 14.1% to 50.7 ± 15.2%, *p* = 0.202, and COR-y: from 67.2 ± 14.0% to 66.2 ± 13.3%, *p* = 0.961, respectively). (Shown in boxplots in Fig. 3 for COR-x and Fig. 4 for COR-y).

### Range of motions

The range of motion (ROM) at the index level decreased from 10.6 ± 4.0° preoperatively to 9.3 ± 4.0° at the early follow-up (*p* = 0.03, the corrected *P* = 0.05 by one-way repeated measures analysis of variance). The ROM at the index level remained unchanged from early follow-up to the final (9.3 ± 4.0° vs 9.5 ± 5.2°, *p* = 0.80). The mean global (C2-7) ROM before surgery was similar to that at early follow-up (46.8 ± 15.2° vs 45.2 ± 13.3°, *p* = 0.46), and it was well preserved during the 10-year follow-up (45.2 ± 13.3° vs 47.3 ± 13.1°, *p* = 0.365).

All radiographic results were summarized in Table 2.

**Table 2:** Radiographic results

Characteristics	Pre-op	Post-op	last Follow-up	*p* value	
				pre-op VS post-op	post-op VS follow-up
Overall ROM (°)	46.8 ± 15.2	45.2 ± 13.3	47.3 ± 13.1	0.46	0.365
Index level ROM (°)	10.6 ± 4.0	9.3 ± 4.0	9.5 ± 5.2	0.03	0.8
C2–7 angle (°)	15.7 ± 10.5	13.9 ± 11.7	14.8 ± 10.7	0.229	0.416
FSU angle (°)	1.2 ± 6.1	1.8 ± 7.1	0.2 ± 6.9	0.268	0.073
FSU height (%)	223.1 ± 12.9	222.2 ± 18.4	220.5 ± 18.2	0.606	0.197
COR-x (%)	42.4 ± 9.7	49.5 ± 14.1	50.7 ± 15.2	< 0.001	0.202
COR-y (%)	70.6 ± 12.7	67.2 ± 14.0	66.2 ± 13.3	0.003	0.961

### Subgroup’s analysis

**Fig. 5:**
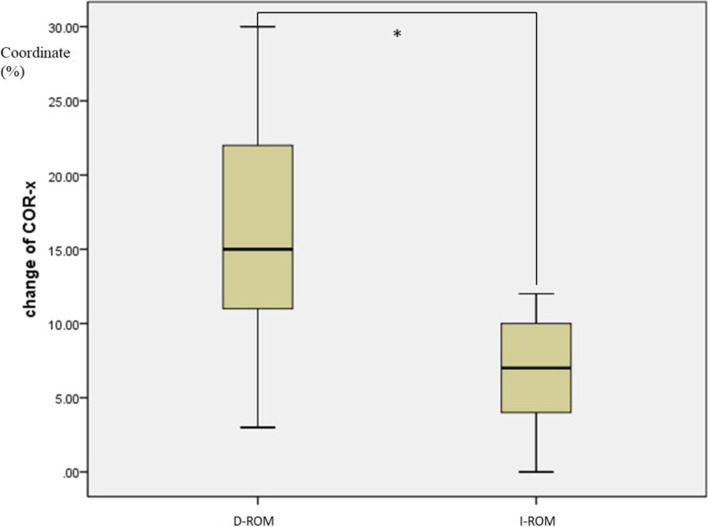
Boxplot shows the comparison of the change of COR-x between two groups (* means significant difference, D-ROM means group with decreased ROM, I-ROM means group with increased ROM)

**Fig. 6:**
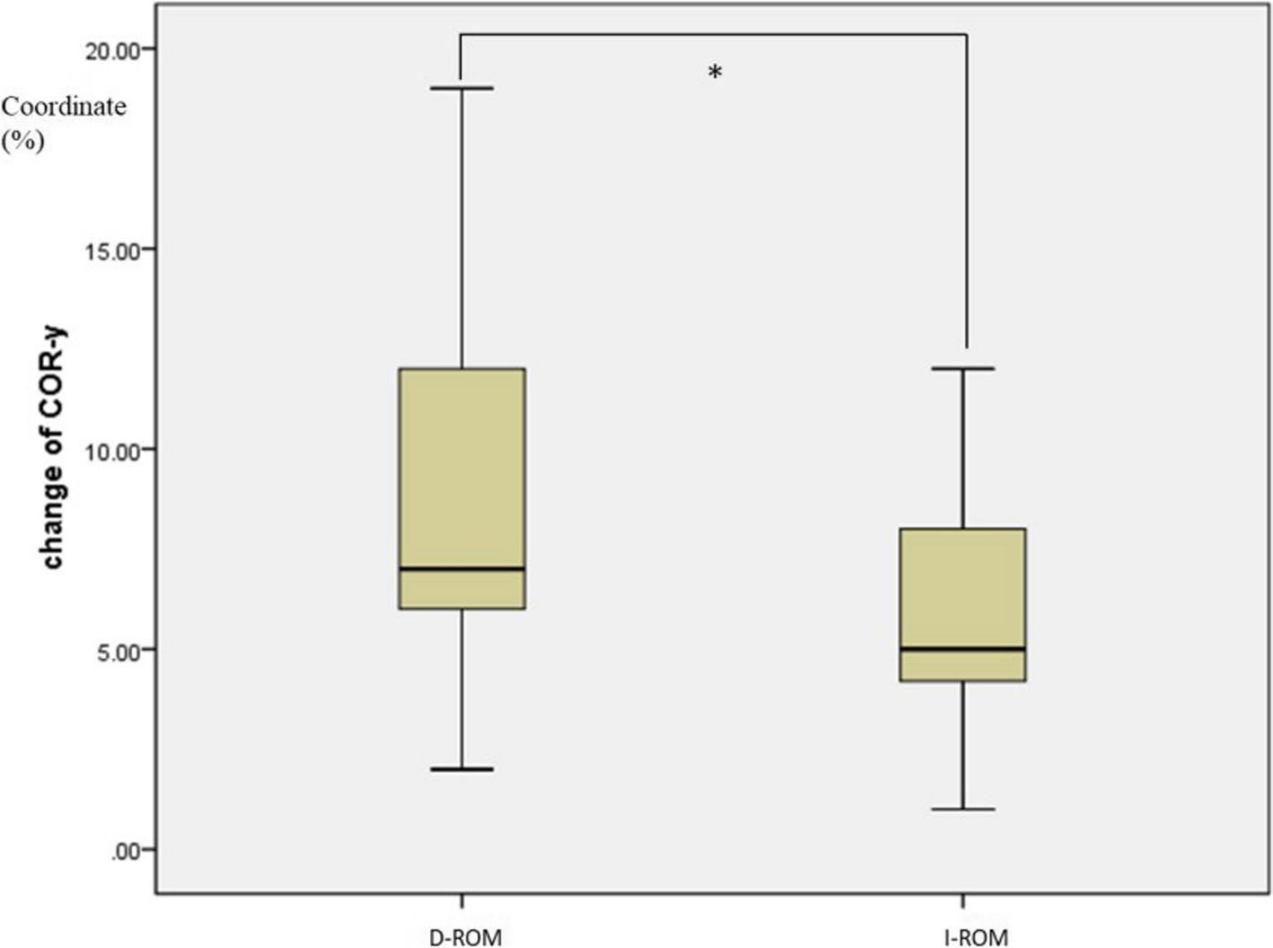
Boxplot shows the comparison of the change of COR-y between two groups (* means significant difference, D-ROM means group with decreased ROM, I-ROM means group with increased ROM)

In D-ROM group, decreased ROM at the final follow-up was found in 31 patients. In I-ROM group, 29 patients showed increased ROM at the index level. There were no significant differences of all baseline characteristics between the two subgroups (Table 3). A comparison of the change of COR-x or COR-y was made between these two groups. The change of COR-x was larger in group D-ROM than that in group I-ROM ( 16.1 ± 7.8 % vs 8.0 ± 5.7 %, *p* < 0.001), which means that COR shifted more anterior in group D-ROM. The change of COR-y was larger in group D-ROM than that in group I-ROM, (8.5 ± 4.2 % vs 5.8 ± 2.6 %, *p* =0.004), indicating that COR in group D-ROM shifted more inferior. (Shown in the boxplots in Fig. 5 for COR-x and Fig. 6 for COR-y).

**Table 3:** Subgroup analysis of baseline characteristics

Characteristics	Subgroup		*p* value
	D-ROM	I-ROM	
Patients (n)	31	29	
Mean age (years)	57.0 ± 9.3	54.7 ± 6.6	0.263
Sex (female/male)	13/18	11/18	0.752^a^
Index level (n)			
C3–4	0	1	0.391^b^
C4–5	9	4	
C5–6	19	20	
C6–7	3	4	
Follow up (months)	135.2 ± 15.4	135.0 ± 16.4	0.975
Overall ROM (°)	47.1 ± 17.4	46.5 ± 12.8	0.879
Index level ROM (°)	11.2 ± 4.0	10.1 ± 3.9	0.275
C2–7 angle (°)	14.0 ± 14.9	11.6 ± 12.9	0.512
FSU angle (°)	2.3 ± 6.7	0.1 ± 5.2	0.131
FSU height (%)	222.3 ± 10.6	224.0 ± 15.1	0.626
COR-x (%)	41.2 ± 9.3	43.5 ± 10.0	0.36
COR-y (%)	69.9 ± 14.0	71.3 ± 11.3	0.673

^a^two-sided χ2 test

^b^two-sided Fisher exact test

## Discussion

The present study focused on the clinical outcome and sagittal kinematics in 60 cases who received Bryan cervical disc arthroplasty with a minimum follow-up of 10 years, and highlighted the influence of the deviated COR on the ROM of the index level.

Cervical disc arthroplasty can achieve favorable clinical and radiographic outcome in previous literature [2, 3, 8, 18, 19]. A meta-analysis indicated that cervical disc arthroplasty was an effective and safe surgical technique, and it was found to be more superior to ACDF in terms of better neurological function, lower neck pain scores at 2 years follow-up [7]. But the follow-up period was relatively short. Badve et al [1] conducted a randomized trial and compared the 10-year outcomes of Bryan CDA versus ACDF. He found that the Bryan disc provided a 12% higher employment rate versus fusion surgery. However, besides the clinical outcome, there was no exploration on the sagittal kinematics. Our data were comparable with that of previous studies. It showed a significant improvement in JOA and NDI scores at the final follow-up, and 91.7% of patients received a good or excellent outcome according to Odom’s scale. Meanwhile, sagittal alignment was well maintained. In addition, we focused on the sagittal kinematics (ROM and COR) after cervical disc arthroplasty for a 10-year follow-up.

ROM is an important variable to assess the mobility of cervical spine. A good residual ROM after cervical disc arthroplasty at last follow-up was reported. Dejaegher et al [10] reported that Mobility of more than 2 degrees after CDA was reached in 81% of patients over a ten-year follow-up. Han et al [9] studied the ROM separating myelopathy from radiculopathy. They found that the ROM of index level was (9.5°± 4.4°) before surgery and maintained at (9.0°± 5.5°) at last follow-up in myelopathy group. And it was (9.5°± 4.6°) and (9.0°± 5.3°) before surgery and at last follow-up in radiculopathy group, respectively. The Bryan prosthesis remained mobile at last follow-up for 78.9% patients in the myelopathy group and 78.6% patients in the radiculopathy group. Among present cases, we noted a minor decrease of ROM of the index level from 10.6° preoperatively to 9.3° at early follow up, and no significantly change was found at final 10-year follow-up. Other kinematic properties of the cervical spine, including C2-C7angle, FSU angle and global ROM were also well maintained.

Theoretically, CDA should not only preserve the quantity of motion like ROM and FSU angle, but also simulate as much as possible the natural quality of cervical spine motion. Thus COR were investigated as, it reflects the quality of motion. Rong et al [17] reported that the mean preoperative location of COR at C5/6 level was located slightly inferior and posterior to the middle of the superior endplate of C6 vertebral body. Amevo [20, 21] and Dvorak [21] both depicted that the distribution of CORs at C5/6 level was approximately at the posterosuperior part of C6 vertebral body. Our study found similar results as the researches above. The inherent COR of the flexion-extension motion before surgery was located in the posterior superior half of the caudal vertebral body, which validated the eligibility of our sample and measurements.

Pickett [6] and Kowalczyk [11] came to the similar conclusion that the Bryan disc did not significantly change the COR coordinates with short-term follow-up (maximal 2 years). While Powell et al [22] found that COR shifted more posterior (1% endplate width) and cephalad (20% endplate width) at the index level compared with the preoperative position after the Bryan cervical disc arthroplasty. On the other hand, we found that the COR shifted downward and forward after CDA, shown by the increased COR-x from a preoperative mean of 42.4±9.7% to 49.5±14.1% at the early follow-up (*p* < 0.001) , and by the decreased COR-y after surgery (70.6 ± 12.7 % vs 67.2 ± 13.9 %, *p* = 0.003). The possible reasons for this deviation may be the more caudal insertion angulation and/or insufficient insertion depth of the artificial disc. Over-milling of the posterosuperior corner of the caudal vertebra during operation may result in caudal insertion angulation of the prosthesis, and this may cause COR deviation. It may warrant detailed studies on that.

Despite of the statistical significance of the anterior-inferior change of COR, the amplitude of variation was relatively small (COR-x: 7.2%, COR-y: 3.4%). Whether this small margin of variation would cause clinical change still needs further researches. But COR-x and COR-y showed no significant change at the last follow-up compared with the early postoperative values (*p* = 0.203 and *p*= 0.109, respectively). It means that the COR remained relatively stable during the 10-year duration after CDA, which strengthened the conclusions of the short-term studies above.

In our subgroup analysis, a deviated COR was related with a decreased ROM. Previous studies found that if the simulated location of COR was close to the inherent location of 1 healthy functional spinal unit, the segmental ROM on the sagittal plane would be well preserved [23, 24]. And in lumbar disc arthroplasty, patients with smaller deviated COR showed increased ROM, while larger deviated COR was related with decreased ROM. Thus, a deviated COR after CDA may be related with a decreased ROM. One functional spinal unit involves one intervertebral joint and two facet joints. The mismatch between intervertebral joint (the artificial disc) and facet joints caused by the deviated COR may lead to decreased ROM. The greater COR changes may be an important risk factor for decreased ROM after cervical disc arthroplasty. However, the specific threshold or cut-off value was still unknown and needs further studies on that.

Limitation exists in our study. Firstly, we used only end flexion-extension images to calculate the mean COR and did not depict the path of the COR from full flexion to full extension. Secondly, all surgeries were performed by a single surgeon. The influence of the surgeon’s preferred technique and experience may not be fully ruled out.

However, our study does have some strengths. First of all, the minimal 10-year follow-up makes our results more stable and solid. Second, we evaluated not only the quantity of the motion-ROM, but also the quality of the motion-COR. Last but not the least, it can provide clinicians some guidance in the preoperative design of cervical disc arthroplasty.

## Conclusion

Our in vivo study illustrated that Bryan cervical disc arthroplasty could achieved favorable clinical results and radiographic outcomes over time. Bryan discs preserved ROM at the index level over a ten-year follow-up. The reduction of the flexion-extension ROM may be correlated with a more deviated postoperative COR, and more attention should be paid to preoperative design and intraoperative technique to obtain a more native COR.

## Data Availability

The data generated and/or analyzed during this study are available from the corresponding author on reasonable request.

## Abbreviations

ACDF: Anterior cervical discectomy and fusion
CDDD: Cervical degenerative disc disease
CDA: Cervical disc arthroplasty
ROM: Range of motion
COR: Center of rotation
FSU: Functional spinal unit
NDI: Neck Disability Index
JOA: Japanese Orthopaedic Association
ICC: Intra-class correlation coefficient
ASD: Adjacent segment disease
